# Exploratory Real-World Observations on Pulmonary Function Evolution, HRCT Patterns, and Antifibrotic Escalation in Systemic Sclerosis-Associated Interstitial Lung Disease Treated with Mycophenolate Mofetil

**DOI:** 10.3390/jcm15114115

**Published:** 2026-05-26

**Authors:** Diana Elena Cosău, Mihai Roca, Alexandru Dan Costache, Irina Iuliana Costache Enache, Ionela Lăcrămioara Șerban, Mara Russu, Vladia Lăpuște, Alexandra Lori Donica, Cristina Pomîrleanu, Codrina Ancuța

**Affiliations:** 1 Faculty of Medicine, “Grigore T. Popa” University of Medicine and Pharmacy, 700115 Iasi, Romania; cosau.diana-elena@d.umfiasi.ro (D.E.C.); adcostache@yahoo.com (A.D.C.); irina.costache@umfiasi.ro (I.I.C.E.); ilserban1@yahoo.com (I.L.Ș.); mara.russu@umfiasi.ro (M.R.); costachescu.alexandra-lori@d.umfiasi.ro (A.L.D.); daniela.pomirleanu@umfiasi.ro (C.P.); codrina.ancuta@umfiasi.ro (C.A.); 2 2nd Rheumatology Department, Clinical Rehabilitation Hospital, 700661 Iasi, Romania; 3 Department of Cardiovascular Rehabilitation, Clinical Rehabilitation Hospital, 700661 Iasi, Romania; 4 Cardiology Clinic, “St. Spiridon” County Emergency Clinical Hospital, 700111 Iasi, Romania

**Keywords:** systemic sclerosis, SSc-ILD, mycophenolate mofetil, nintedanib, interstitial lung disease, UIP pattern, NSIP pattern, DLCO, pulmonary function, disease progression

## Abstract

Systemic sclerosis-associated interstitial lung disease (SSc-ILD) remains an important cause of morbidity and mortality in systemic sclerosis. Mycophenolate mofetil (MMF) is widely used as first-line immunosuppressive therapy; however, real-world descriptions of pulmonary functional and radiologic evolution during MMF therapy remain limited, particularly according to high-resolution computed tomography (HRCT) pattern. **Objectives:** To descriptively evaluate pulmonary function evolution, radiologic findings, and safety outcomes in patients with SSc-ILD treated with MMF in routine clinical practice, with exploratory analyses according to HRCT pattern and subsequent antifibrotic use. **Materials and Methods:** We conducted a retrospective single-center study including 20 patients with SSc-ILD treated with MMF. Clinical, functional (forced vital capacity [FVC]; diffusing capacity for carbon monoxide [DLCO]), and radiologic (HRCT Warrick score) parameters were assessed at baseline, 6 months, and 12 months. Patients were stratified according to nonspecific interstitial pneumonia (NSIP) and usual interstitial pneumonia (UIP) patterns. Statistical analyses were exploratory and descriptive. **Results:** Pulmonary function remained overall stable during follow-up under MMF therapy, while DLCO improvement was observed at 6 months and remained stable at 12 months. Radiologic progression appeared more limited in patients with NSIP pattern, whereas patients with UIP pattern generally exhibited more frequent radiologic progression during follow-up. Patients who subsequently received nintedanib generally presented with UIP-pattern disease, lower baseline DLCO values, and more advanced pulmonary involvement. MMF was generally well tolerated, with treatment discontinuation due to adverse events observed in a single patient. **Conclusions:** This small retrospective real-world case series describes pulmonary functional and radiologic evolution in patients with SSc-ILD treated with MMF in routine clinical practice. Overall functional stabilization was observed during follow-up, while radiologic progression was more frequently observed in patients with UIP-pattern disease and more advanced baseline pulmonary involvement. Because of the exploratory descriptive design and limited sample size, these observations should be interpreted cautiously and considered hypothesis-generating only. Further prospective studies with standardized radiologic assessment are required.

## 1. Introduction

Systemic sclerosis (SSc) is a complex autoimmune connective tissue disease characterized by immune dysregulation, microvascular injury, and progressive fibrosis affecting the skin and internal organs [1]. Among its visceral manifestations, interstitial lung disease (ILD) represents one of the most frequent and clinically significant complications, being a leading cause of morbidity and mortality in patients with SSc [2].

SSc-associated ILD (SSc-ILD) exhibits marked heterogeneity in both clinical presentation and disease trajectory, ranging from stable or slowly progressive disease forms with minimal functional impairment to rapidly progressive fibrosing phenotypes associated with significant functional decline and poor prognosis [2].

High-resolution computed tomography (HRCT) plays a central role in disease characterization, with nonspecific interstitial pneumonia (NSIP) representing the most frequent pattern and generally associated with a more favorable outcome, whereas usual interstitial pneumonia (UIP), although less common, has been described in previous studies as being associated with more advanced fibrosing disease and less favorable pulmonary evolution [3,4,5].

The therapeutic landscape of SSc-ILD has evolved significantly in recent years. Mycophenolate mofetil (MMF) has emerged as a cornerstone of first-line immunosuppressive therapy, supported by randomized clinical trials showing associations with stabilization or modest improvement of lung function, together with a favorable safety profile compared to cyclophosphamide [6,7,8]. Nevertheless, treatment response remains variable, and a substantial proportion of patients continue to experience disease progression despite immunosuppressive therapy [6,7,8,9,10,11]. This apparent discrepancy raises an important clinical and pathophysiological question regarding the extent to which MMF influences the different components of SSc-ILD. While functional parameters such as forced vital capacity (FVC) and diffusing capacity for carbon monoxide (DLCO) may stabilize or improve, radiologic progression—reflecting ongoing fibrotic remodeling—may still occur, particularly in patients with a UIP pattern. This dissociation between functional stabilization and structural progression has been described in previous studies and may also be observed in routine clinical practice.

The introduction of antifibrotic agents, particularly nintedanib, has further expanded therapeutic options for SSc-ILD. In routine clinical practice, combined immunosuppressive and antifibrotic approaches are increasingly used in patients with progressive fibrosing disease. However, real-world descriptions of pulmonary evolution, radiologic progression, and subsequent antifibrotic initiation during MMF therapy remain limited, particularly according to HRCT pattern [9,12].

In this context, real-world data may complement evidence from randomized clinical trials by reflecting the heterogeneity of patients, treatment pathways, and monitoring strategies encountered in routine practice. Although MMF is already established as standard first-line therapy in SSc-ILD, less information is available regarding the real-world evolution of pulmonary function, radiologic findings, and subsequent antifibrotic use during MMF therapy.

Although current ATS/EULAR recommendations already support the use of MMF and antifibrotic therapy in SSc-ILD, less information is available regarding how MMF and antifibrotic therapies are used in routine clinical practice, particularly in relation to radiologic progression, treatment sequencing, and subsequent antifibrotic use across different HRCT patterns. The present study was designed to provide exploratory real-world observations addressing these aspects within a retrospective single-center cohort.

Therefore, the present study aimed to descriptively evaluate pulmonary function evolution, radiologic findings, and subsequent antifibrotic use in patients with SSc-ILD treated with MMF in routine clinical practice, with exploratory analyses according to HRCT pattern (NSIP versus UIP).

## 2. Materials and Methods

### 2.1. Study Design and Population

This retrospective, single-center observational study was conducted in a tertiary academic center within the Rheumatology Department of the Clinical Rehabilitation Hospital, Iași, Romania.

Among the 60 patients with SSc routinely followed in this department, a subset was diagnosed with SSc-ILD, based on HRCT and pulmonary function testing. Of these, 20 patients were receiving treatment with MMF and were included in the present analysis.

Patients were retrospectively evaluated over a 24-month period (December 2023 to December 2025), reflecting real-world clinical practice.

**Figure 1:**
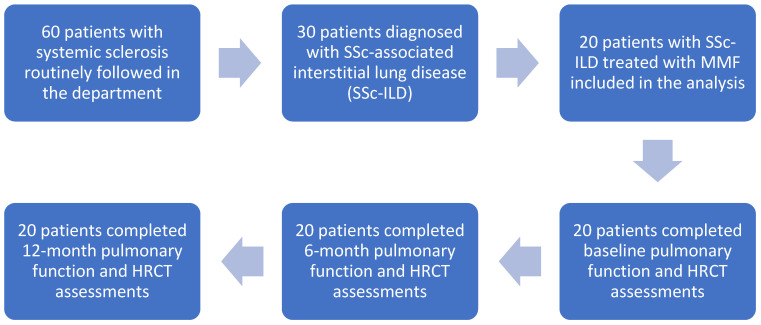
Flow diagram summarizing patient selection and completion of longitudinal pulmonary function and HRCT assessments during follow-u.

A flow diagram summarizing patient selection and completion of longitudinal follow-up assessments is presented in Figure 1.

### 2.2. Data Collection and Variables

Data were retrospectively extracted from medical records and included demographic characteristics, disease-related variables, pulmonary function parameters, and imaging findings.

Pulmonary involvement was assessed using FVC (% predicted) and DLCO (% predicted) (COSMED pulmonary function testing system, COSMED Srl, Rome, Italy; Functional Department, Clinical Rehabilitation Hospital), measured at baseline, 6 months, and 12 months when available, irrespective of clinical presentation (asymptomatic, dyspnea, cough, Velcro crackles).

Radiologic evaluation was performed using HRCT (Philips Brilliance 3500 CT scanner, Philips Healthcare, Amsterdam, The Netherlands; Radiological Department, Clinical Rehabilitation Hospital), with assessment of both pattern and extent of interstitial lung disease. HRCT examinations were performed as part of routine clinical care using standard thoracic imaging acquisition protocols routinely applied in the institution for the evaluation of interstitial lung disease. Imaging studies were retrospectively reviewed through formal radiologic reports documented in the medical records.

The predominant HRCT pattern was classified as either non-specific interstitial pneumonia (NSIP) or usual interstitial pneumonia (UIP), according to standard radiologic criteria as documented in formal radiology reports [3]. The extent and severity of pulmonary involvement were quantified using the Warrick score (range 0–30), categorized as mild (0–8), moderate (9–15), or severe (>15). The Warrick score is a semi-quantitative HRCT scoring system designed to evaluate both the severity and extent of interstitial lung abnormalities in connective tissue disease-associated interstitial lung disease. The score incorporates several radiologic abnormalities, including ground-glass opacities, irregular pleural margins, septal and subpleural lines, honeycombing, and subpleural cysts, with higher scores reflecting more extensive fibrotic lung involvement and greater structural pulmonary damage [13].

Radiologic assessment was based on routine clinical HRCT reports and semi-quantitative Warrick scoring performed in real-world clinical practice, without centralized or blinded radiologic review. HRCT examinations were interpreted by radiologists involved in routine patient care using standard institutional thoracic imaging protocols. Because imaging evaluations were performed retrospectively in routine clinical settings, formal interobserver variability assessment was not available and some degree of interobserver variability cannot be excluded.

Interstitial lung disease progression was defined in accordance with criteria for progressive fibrosing ILD, as any of the following: (i) a relative decline in FVC ≥ 10% from baseline; (ii) a decline in FVC of 5–9% associated with clinical and/or radiologic worsening; or (iii) radiologic progression on HRCT. These criteria were applied in a pragmatic manner consistent with routine clinical practice [1,9,14,15].

Pulmonary disease severity was assessed using the pulmonary component of the Medsger severity scale, while overall organ involvement was evaluated using the same instrument [14]. Nailfold capillaroscopy findings were classified as early, active, or late scleroderma patterns [16].

The primary outcome was the evolution of pulmonary function under MMF therapy, assessed by changes in FVC and DLCO over time. Secondary outcomes included radiologic progression (Warrick score), changes in pulmonary disease severity (Medsger scale), and subsequent initiation of nintedanib during follow-up. Safety outcomes included adverse events and treatment discontinuation related to MMF.

### 2.3. Statistical Analysis

Continuous variables are presented as mean ± standard deviation or median (interquartile range), depending on distribution, while categorical variables are expressed as absolute numbers and percentages. Comparisons between baseline and follow-up values were performed using paired *t* test or the Wilcoxon signed-rank test, as appropriate. Given the limited sample size and substantial variability across measurements, all statistical analyses were considered exploratory, and *p*-values were interpreted descriptively rather than as evidence of definitive associations or treatment effects. Accordingly, effect estimates (mean differences) together with 95% confidence intervals were additionally reported to facilitate interpretation of the magnitude and precision of observed differences.

Subgroup analyses were conducted according to HRCT pattern (NSIP versus UIP) to explore differences in functional evolution, radiologic progression, and subsequent initiation of nintedanib.

Additionally, an exploratory adjusted logistic regression analysis was performed to assess factors associated with subsequent initiation of nintedanib. Due to the limited sample size and low number of events, only clinically relevant variables (HRCT pattern and baseline DLCO) were included in the model to reduce the risk of overfitting. Accordingly, all subgroup and regression analyses should be interpreted as exploratory and descriptive rather than confirmatory. Because of the exploratory nature of the study and the limited cohort size, the regression analysis was not intended to establish independent associations or causal relationships, but rather to explore potential associations requiring validation in larger prospective studies.

Complete pulmonary function and HRCT follow-up assessments were available for all included patients at baseline, 6 months, and 12 months; therefore, no imputation procedures were required.

All statistical analyses were performed using IBM SPSS Statistics software version 26.0 (IBM Corp., Armonk, NY, USA).

## 3. Results

All 20 included patients completed the scheduled pulmonary function and HRCT evaluations at baseline, 6 months, and 12 months.

### 3.1. Baseline Characteristics

The demographic, clinical, serological, functional, and imaging characteristics of the study population are summarized in Table 1.

The mean age was 56.4 ± 11.1 years, with a predominance of female sex (75%). Most patients had diffuse cutaneous SSc (65%), while limited cutaneous SSc and scleroderma sine scleroderma were less frequent.

HRCT evaluation revealed a predominance of the NSIP pattern (65%), while UIP was identified in 35% of patients. The extent and severity of pulmonary involvement, assessed using the Warrick score, indicated predominantly moderate-to-severe interstitial fibrosis across the cohort, reflecting a substantial structural lung disease burden at baseline.

At baseline, patients with UIP exhibited a higher radiologic burden, reflected by increased Warrick scores and more advanced pulmonary involvement compared to those with NSIP. These baseline differences suggest that patients with UIP-pattern disease may already have represented a subgroup with more advanced pulmonary involvement at study entry. Consequently, subsequent radiologic progression and antifibrotic escalation observed during follow-up may partially reflect greater baseline disease severity rather than the isolated effect of HRCT pattern alone.

Pulmonary involvement was frequently advanced, with 55% of patients classified as having severe disease according to the Medsger pulmonary score (score 3–4); mild disease (scores 0–1) was observed in 3 patients (15%), moderate involvement (score 2) in 6 patients (30%). This distribution highlights that interstitial lung disease was not only prevalent but frequently advanced at the time of evaluation, corresponding with markedly reduced DLCO and, in some cases, the need for supplemental oxygen.

From a functional perspective, mean FVC was relatively preserved (91.2% predicted), whereas DLCO was markedly reduced (31.4% predicted), indicating significant impairment in gas exchange despite maintained lung volumes.

Serologically, anti-Scl-70 antibodies were present in 85% of patients, consistent with a fibrosing phenotype, while anticentromere antibodies were detected in 15%.

Multisystem involvement was common, including cutaneous (75%) (mean modified Rodnan skin score of 18, frequently associated with digital ulcers and telangiectasias); cardiac (50%) (heart failure, arrhythmias, valvular regurgitation); gastrointestinal (30%) (dysphagia and gastroesophageal reflux disease); and renal (20%) manifestations (mild impairment, with a mean proteinuria of 300 mg/24 h and an estimated glomerular filtration rate of 65 mL/min/1.73 m^2^).

Nailfold capillaroscopy demonstrated a predominance of the active scleroderma pattern (65%), indicating ongoing microvascular injury, while early and late patterns were observed in 10% and 15% of cases, respectively.

Overall, these findings indicate a cohort characterized by advanced pulmonary involvement, high fibrotic burden, and active systemic disease at baseline.

### 3.2. Primary Outcome: Evolution of Pulmonary Function Under MMF

**Figure 2:**
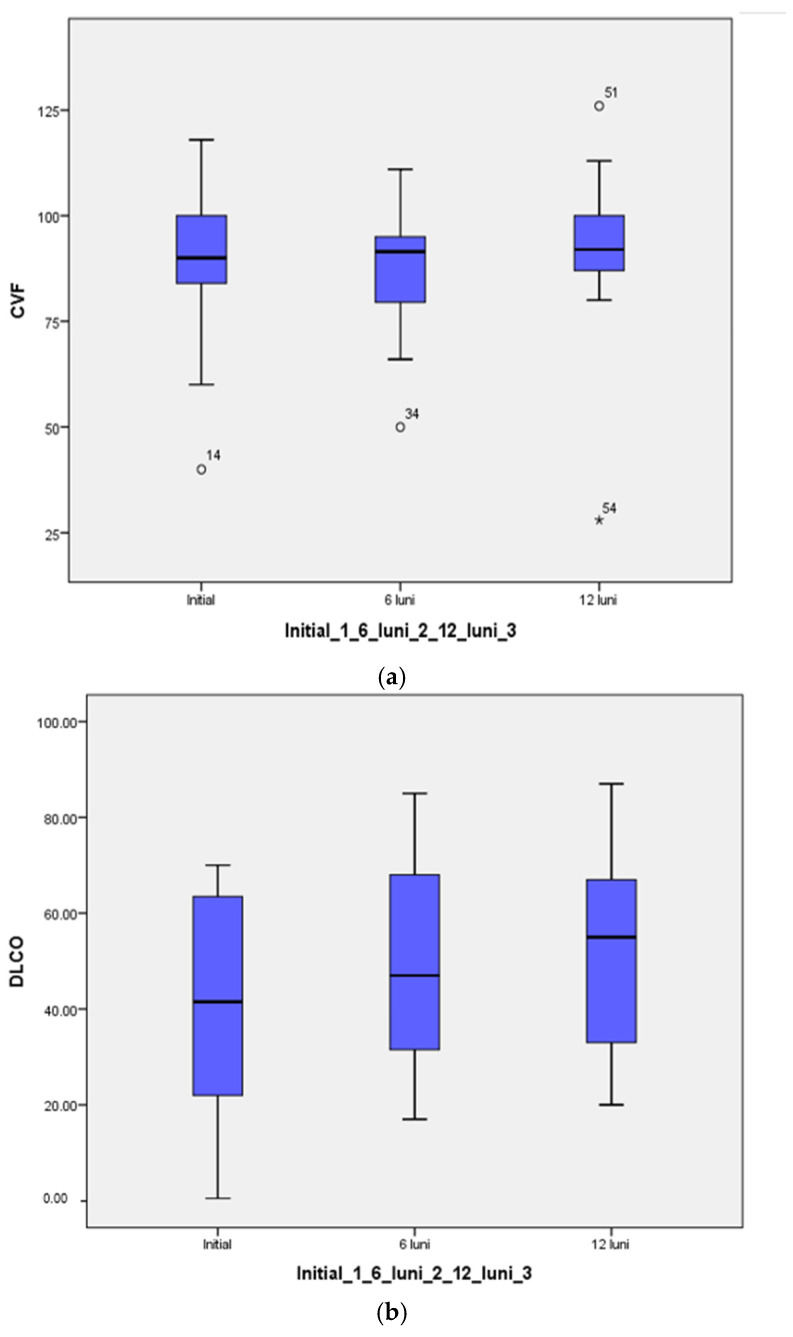
Evolution of pulmonary function under MMF therapy: (**a**) FVC and (**b**) DLCO at baseline, 6 months, and 12 months.

The evolution of FVC and DLCO under MMF therapy is presented in Table 2 and illustrated in Figure 2a,b.

#### 3.2.1. Evolution of FVC

At the individual level, 17 of 20 patients (85%) demonstrated stable or increased FVC over 12 months, while 3 patients (15%) met criteria for functional progression, descriptively suggesting relative stability of ventilatory function during follow-up under MMF therapy (Figure 2a).

At the group level, comparison of baseline FVC values with those at 6 months showed no statistically significant change (mean difference +0.8%, 95% CI −4.34 to 5.94; *p* = 0.748), indicating preservation of ventilatory function in the short term. Similarly, no significant difference was observed between 6 and 12 months (mean difference +0.7%, 95% CI −6.34 to 7.73; *p* = 0.834).

Comparison between baseline and 12-month FVC values showed a numerical increase that did not reach conventional statistical significance (mean difference +6.3%, 95% CI −0.06 to 12.68; *p* = 0.052) (Table 2).

Given the exploratory nature of the study and limited sample size, these findings should be interpreted cautiously as descriptive observations rather than evidence of definitive treatment effect.

#### 3.2.2. Evolution of DLCO

DLCO showed a numerical improvement at 6 months compared with baseline (mean difference +5.8%, 95% CI 0.07 to 11.58; *p* = 0.048), reaching nominal statistical significance and descriptively suggesting an early improvement in gas exchange during follow-up under MMF therapy. This improvement was sustained at 12 months compared with baseline (mean difference +9.0%, 95% CI 1.61 to 16.39; *p* = 0.021), whereas no significant difference was observed between 6 and 12 months (mean difference +3.1%, 95% CI −2.72 to 8.86; *p* = 0.272) (Table 2), suggesting stabilization after the initial improvement phase.

Overall, DLCO descriptively showed an early numerical improvement followed by relative stabilization during follow-up.

### 3.3. Secondary Outcome: Radiologic Progression (Warrick Score)

**Figure 3:**
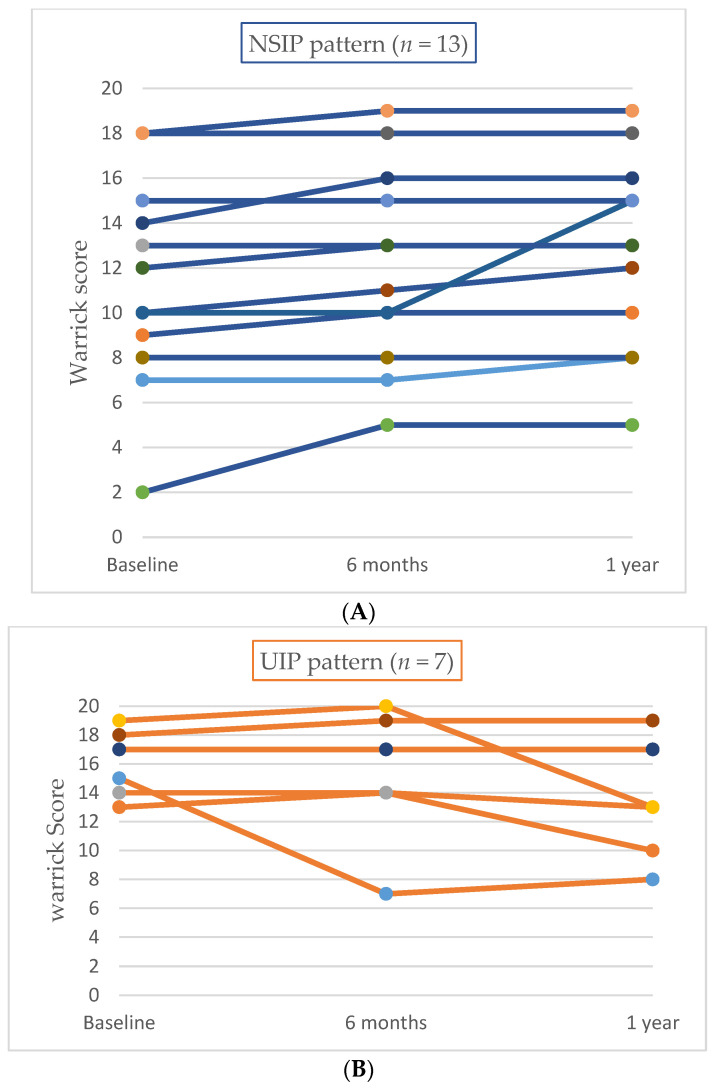
Individual patient-level trajectories of Warrick score during MMF therapy according to HRCT pattern. Each line represents an individual patient trajectory. (**A**) NSIP pattern (*n* = 13); (**B**) UIP pattern (*n* = 7).

Radiologic evolution assessed by the Warrick score is illustrated in Figure 3.

When analyzed according to HRCT pattern, patients with NSIP exhibited lower baseline Warrick scores and a modest increase over time. In contrast, patients with UIP showed higher baseline scores and a more pronounced increase during follow-up.

These observations descriptively suggest that radiologic progression may still be observed despite apparent functional stabilization, particularly in patients with UIP pattern. However, these imaging findings should be interpreted cautiously given that HRCT evaluation was based on routine clinical reporting and semi-quantitative scoring without centralized blinded imaging review.

### 3.4. Secondary Outcome: Disease Severity (Medsger Score Evolution)

**Figure 4:**
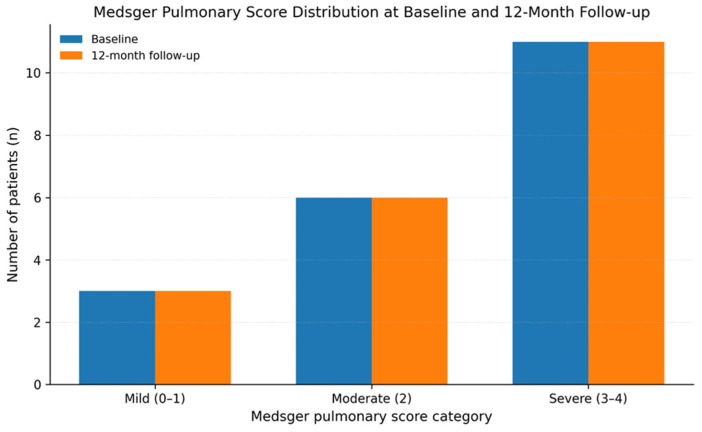
Distribution of Medsger Pulmonary Severity Scores at baseline and 12 months under MMF therapy.

The distribution of pulmonary disease severity according to the Medsger scale at baseline and 12 months is illustrated in Figure 4.

No major shifts in severity categories were observed over time, suggesting overall stabilization of disease severity under MMF therapy. However, the persistence of moderate-to-severe disease in the majority of patients reflects the chronic and progressive nature of SSc-ILD.

Most patients presented with moderate-to-severe pulmonary involvement at baseline. No major shifts in severity categories were observed at 12 months, indicating overall stabilization of disease severity under MMF therapy, despite the high baseline burden of interstitial lung disease.

### 3.5. Descriptive Observations According to Subsequent Nintedanib Use

Descriptive characteristics of patients who subsequently received nintedanib are presented in Table 3a while exploratory adjusted analyses are summarized in Table 3b.

During follow-up, 11 of 20 patients (55%) subsequently initiated antifibrotic therapy with nintedanib, while 9 patients (45%) did not.

Patients who subsequently initiated nintedanib generally presented with more severe pulmonary involvement at baseline, including lower DLCO values, a higher prevalence of severe ILD, and a predominance of the UIP pattern. A lower baseline DLCO was observed in the nintedanib group (40 [35–40] vs. 60 [55–60], *p* = 0.04), suggesting greater impairment in gas exchange at study entry.

In addition, the presence of a UIP pattern on HRCT was more frequent among patients who subsequently received antifibrotic therapy (7/11 patients, 63.6% vs. 0/9, 0%; *p* = 0.01), whereas all patients who did not receive nintedanib had an NSIP pattern (100%, *p* = 0.01).

Severe ILD, defined by DLCO < 40%, was also more frequent in patients who subsequently initiated nintedanib (6/11, 54.5% vs. 1/9, 11.1%; *p* = 0.03), descriptively suggesting that patients who later received nintedanib had more advanced pulmonary involvement at baseline.

Although not reaching statistical significance, patients in the nintedanib group tended to have longer disease duration (approximately 9–10 vs. 2–3 years, *p* = 0.06) and a higher prevalence of diffuse cutaneous systemic sclerosis (81.8% vs. 44.4%, *p* = 0.09), suggesting a trend toward a more advanced disease phenotype.

No significant differences were observed in age (58 ± 10 vs. 53 ± 11 years, *p* = 0.28), sex distribution (72.7% vs. 77.8% female, *p* = 1.00), FVC values (85% vs. 95%, *p* = 0.20), or dyspnea prevalence (90.9% vs. 77.8%, *p* = 0.58).

However, initiation of nintedanib was based on physician decision-making in routine clinical practice and may also have been influenced by local prescribing practices, drug availability, reimbursement criteria, and individual clinical judgment.

#### Supplementary Exploratory Regression Analysis

An exploratory adjusted logistic regression analysis including HRCT pattern and baseline DLCO was additionally performed. UIP pattern showed a non-significant trend toward association with subsequent nintedanib initiation (adjusted OR 3.23, 95% CI 0.41–25.23, *p* = 0.263), while baseline DLCO was not significantly associated with subsequent antifibrotic initiation after adjustment (adjusted OR 0.97, 95% CI 0.91–1.03, *p* = 0.297).

Given the limited sample size and low number of events, the adjusted model was intentionally restricted to two clinically relevant variables in order to reduce the risk of overfitting and preserve model stability. Consequently, these analyses should be interpreted as exploratory and descriptive rather than confirmatory.

Because of the very small cohort size and low number of events, this supplementary exploratory model was performed only to address potential collinearity among severity variables and should not be interpreted as evidence of independent predictors or causal associations.

### 3.6. Correlation Between Warrick Score and DLCO

**Figure 5:**
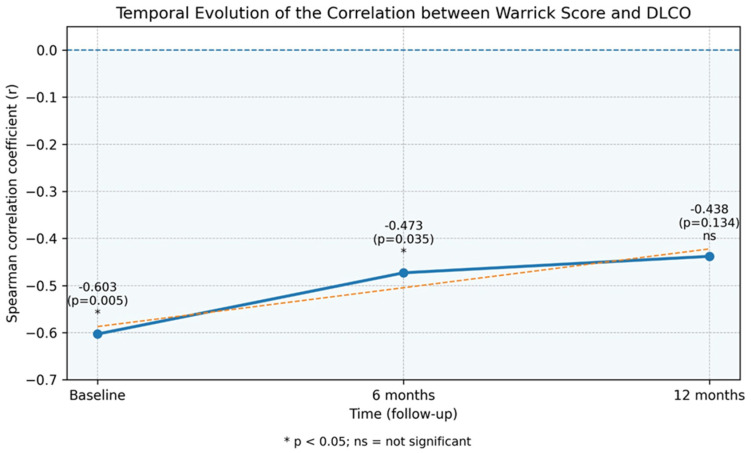
Temporal dynamics of the correlation between Warrick score and DLCO.

The correlation between Warrick score and DLCO was assessed at baseline, 6 months, and 12 months using Spearman correlation analysis. A moderate inverse correlation was observed at baseline (ρ = −0.603, *p* = 0.005) and at 6 months (ρ = −0.473, *p* = 0.035), both reaching statistical significance. At 12 months, the correlation remained negative (ρ = −0.438) but did not reach statistical significance (*p* = 0.134). The temporal dynamics of this association are illustrated in Figure 5.

### 3.7. Treatment Characteristics of MMF

Treatment initiation characteristics are detailed in Table 4. Mycophenolate mofetil was used as first-line therapy in 7 patients (35%), while the majority (13 patients, 65%) received MMF as second-line or subsequent therapy following prior immunosuppressive or symptomatic treatments, including methotrexate, azathioprine, hydroxychloroquine, cyclophosphamide, or vasodilator therapy. Details regarding prior therapeutic exposure before MMF initiation are summarized in Table 4.

The indications for MMF initiation were heterogeneous, most commonly reflecting progressive interstitial lung disease with functional decline (particularly reduced DLCO), more advanced pulmonary involvement, or inadequate response or intolerance to previous therapies. In a subset of patients, MMF was initiated early after diagnosis due to extensive pulmonary involvement or clinically concerning disease features at presentation. Because treatment strategies reflected routine real-world clinical practice, patients exhibited heterogeneous therapeutic histories and variable prior immunosuppressive exposure before MMF initiation.

The time from systemic sclerosis diagnosis to MMF initiation showed considerable variability, with a median of 3 years (IQR 0.85–6.5) and a mean of 4.9 years, ranging from 0.5 to 18 years. Early initiation (<1 year) was observed in 25% of patients, whereas 40% initiated treatment between 1 and 5 years and 35% after more than 5 years, reflecting variability in disease course and management approaches observed in routine clinical practice. Because of the retrospective design, cumulative exposure to previous therapies and formal washout periods before MMF initiation could not be systematically standardized or fully evaluated.

Because most patients received MMF after prior exposure to other immunosuppressive therapies, the observed pulmonary evolution during follow-up likely reflects the cumulative effect of sequential therapeutic exposures rather than the isolated effect of MMF alone. Patients initiating MMF as subsequent-line therapy generally exhibited more advanced pulmonary involvement, which may also have influenced subsequent functional and radiologic outcomes.

### 3.8. Descriptive Characteristics According to Timing of MMF Initiation

We descriptively evaluated clinical characteristics according to timing of MMF initiation (Table 5). The only variable significantly associated with treatment timing was the presence of diffuse cutaneous systemic sclerosis, which was more frequent among patients receiving MMF as subsequent-line therapy compared to first-line use (84.6% vs. 28.6%, *p* = 0.03). Diffuse cutaneous SSc was more frequently observed among patients receiving MMF as subsequent-line therapy. Patients receiving subsequent-line MMF generally exhibited longer disease duration and more advanced systemic disease characteristics during routine clinical care.

Patients in the subsequent MMF group had a longer disease duration at treatment initiation (10.7 ± 15.8 vs. 1.8 ± 2.3 years), although this difference did not reach statistical significance (*p* = 0.09), possibly reflecting delayed escalation or prior use of alternative immunosuppressive therapies. No other variables, including age, sex, pulmonary function parameters, dyspnea, ILD pattern, or prevalence of severe ILD, were significantly associated with treatment timing.

### 3.9. Safety and Tolerability of MMF

**Figure 6:**
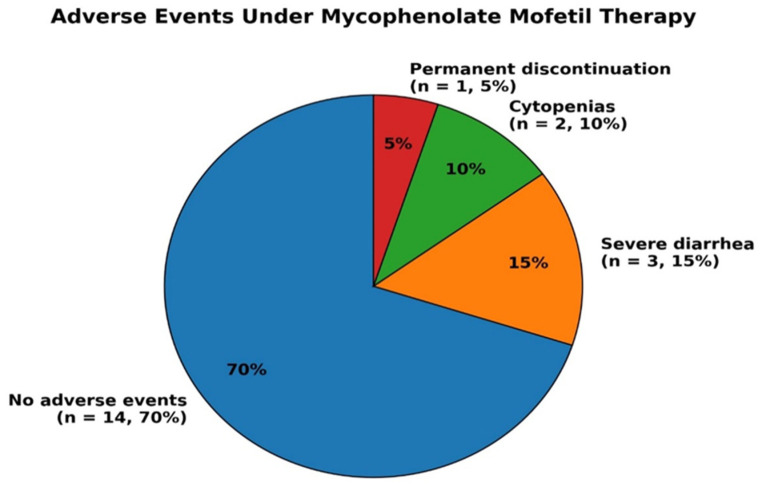
Tolerability and adverse events associated with MMF therapy in SSc-ILD patients.

Safety outcomes are illustrated in Figure 6.

MMF was generally well tolerated. Fourteen patients (70%) did not report any adverse events. Three cases of severe diarrhea (15%) were recorded, requiring dose adjustment and supportive symptomatic management. In addition, two cases of cytopenias (10%), manifested as leukopenia and/or anemia, were documented, necessitating close monitoring and reduction in the MMF dose.

Permanent discontinuation of MMF occurred in a single patient (5%).

Overall, these findings confirm a favorable safety profile of MMF, with a low rate of treatment discontinuation.

## 4. Discussion

The comparison between DLCO values at 6 and 12 months did not reveal a statistically significant difference (*p* = 0.272), suggesting that the improvement in gas exchange observed during follow-up under MMF therapy occurs early after treatment initiation and is subsequently maintained over time. This pattern was more evident in patients with an NSIP pattern, who also demonstrated a more favorable clinical course.

In contrast, patients with a UIP pattern exhibited a higher radiologic burden at baseline, reflected by increased Warrick scores, which continued to rise during follow-up, indicating persistent radiologic progression during follow-up despite MMF therapy. These findings suggest that while functional stabilization was observed under MMF therapy, radiologic progression may still occur, particularly in patients with a UIP pattern, in whom more frequent radiologic progression was observed during follow-up. Importantly, interpretation of these observations should consider that patients with UIP pattern also presented with more advanced pulmonary involvement at baseline, including higher Warrick scores and more severe impairment in gas exchange.

Therefore, the more frequent radiologic progression during follow-up and subsequent antifibrotic use observed in the UIP subgroup may reflect greater baseline disease severity, in addition to potential pattern-specific differences in disease behavior. Because of the limited subgroup size, the present study cannot disentangle the independent contribution of HRCT pattern from that of baseline disease severity.

However, given the exploratory design and limited sample size, these observations should be interpreted cautiously and cannot support definitive pattern-based therapeutic recommendations. Moreover, the very small subgroup numbers increase the possibility of unstable estimates and limit the robustness of comparisons between UIP and NSIP patterns.

Our data also highlight variability in treatment initiation, with MMF frequently introduced as second-line therapy, often several years after diagnosis, particularly in patients with more severe or progressive disease. This delay may reflect the complexity and heterogeneity of treatment pathways in real-world clinical practice and illustrates variability in the timing of MMF initiation observed in routine clinical practice.

Importantly, both stratified and exploratory adjusted analyses suggested that patients who subsequently received nintedanib generally presented with more advanced pulmonary involvement at baseline, including lower DLCO values, UIP pattern, and severe ILD. Although not statistically significant, longer disease duration and a higher prevalence of diffuse cutaneous SSc were also observed in this group, supporting the concept that these observations descriptively reflect that patients who subsequently received nintedanib generally exhibited more advanced pulmonary disease during routine clinical care.

Nevertheless, these associations should be interpreted with caution because the study was not powered to identify independent associations with progression or subsequent antifibrotic use. The small sample size, limited subgroup numbers, and exploratory nature of the analyses substantially restrict the strength and generalizability of these observations.

The adjusted regression model was intentionally restricted to a limited number of clinically relevant variables to reduce the risk of overfitting; therefore, robust associations cannot be definitively established. Consequently, the observed associations should be regarded as exploratory rather than confirmatory.

Overall, these findings provide descriptive real-world observations regarding pulmonary functional and radiologic evolution during MMF therapy in patients with SSc-ILD. However, larger prospective studies are required before these observations can be further evaluated in larger prospective studies. Rather than attempting to redefine current therapeutic paradigms or established prognostic factors in SSc-ILD, this study should be interpreted as complementary to existing guideline-based evidence. Its principal contribution lies in describing exploratory real-world observations regarding treatment patterns, radiologic-functional evolution, and subsequent antifibrotic use in a small retrospective cohort of patients with SSc-ILD managed in routine clinical practice.

The present study does not aim to challenge current therapeutic recommendations or redefine established prognostic factors in SSc-ILD. Rather, it provides exploratory real-world observations regarding the relationship between pulmonary function evolution, radiologic progression, and pulmonary functional and radiologic evolution under MMF therapy.

The main observations of this cohort can be summarized as follows: (i) forced vital capacity remained overall stable over 12 months, with a trend toward improvement; (ii) diffusing capacity for carbon monoxide showed an early improvement that was maintained over time; (iii) radiologic progression was still observed despite therapy, particularly in patients with a usual interstitial pneumonia pattern; (iv) patients who subsequently received nintedanib generally exhibited UIP-pattern disease and more advanced pulmonary impairment at baseline.

Taken together, these results suggest that MMF therapy was associated with functional stabilization but persistent radiologic progression was still observed in some patients during follow-up. These findings should be regarded as exploratory real-world observations that require confirmation in larger, prospective, and standardized studies before these observations can be better understood.

### 4.1. Functional Response Under MMF: Stabilization Rather than Reversal

The observed stabilization of FVC during follow-up is consistent with the known mechanism of action of MMF as an immunomodulatory agent that primarily attenuates inflammation-driven injury rather than reversing established fibrosis. Although FVC remained overall stable during follow-up, the observed numerical increase at 12 months did not reach conventional statistical significance. Therefore, these findings should be interpreted cautiously as exploratory observations rather than evidence of definitive functional improvement under MMF therapy.

In contrast, DLCO showed a statistically significant improvement at 6 months that was maintained at 12 months. An early improvement in alveolar–capillary function was observed during follow-up under MMF therapy, potentially reflecting changes in inflammatory activity and microvascular involvement. No significant difference was observed between 6 and 12 months, suggesting stabilization of DLCO after the initial improvement phase.

Importantly, the combination of stable FVC and improved DLCO is consistent with overall disease stabilization observed during follow-up under MMF therapy rather than reversal of established fibrotic disease.

However, interpretation of treatment response should take into account that the majority of patients in the present cohort had previous exposure to other immunosuppressive therapies before MMF initiation. Consequently, the observed stabilization of pulmonary function cannot be attributed exclusively to MMF, as cumulative effects of prior and sequential therapies may have contributed to the clinical evolution observed during follow-up.

### 4.2. Exploratory Differences According to HRCT Pattern

The present cohort showed exploratory differences in disease evolution according to HRCT pattern under MMF therapy. Patients with an NSIP pattern appeared to exhibit a more favorable clinical course, characterized by relative preservation of lung function and slower radiologic progression. In contrast, patients with a UIP pattern presented with more severe disease at baseline and more frequent functional decline and radiologic worsening during follow-up.

These findings are consistent with previous reports indicating that UIP-pattern disease in this cohort was descriptively associated with more advanced pulmonary involvement and more frequent progression during follow-up despite immunosuppressive therapy.

The persistent increase in Warrick scores in UIP patients, despite MMF treatment, is consistent with the possibility of ongoing fibrotic progression in this subgroup.

However, patients with UIP pattern already demonstrated more frequent radiologic progression and more severe pulmonary impairment at baseline. Consequently, the observed differences in progression during follow-up may not exclusively reflect differences related to HRCT pattern, but could also be influenced by more advanced disease severity at study entry. Given the limited number of UIP patients included in the cohort, the present study was not sufficiently powered to determine whether HRCT pattern was independently associated with progression beyond baseline functional and radiologic severity.

Overall, these observations descriptively reflect differences in pulmonary evolution according to HRCT pattern within this small retrospective cohort. Nevertheless, these observations should be considered exploratory and require confirmation in larger prospective studies.

Importantly, the observation of more frequent progression during follow-up in UIP-pattern disease is consistent with existing literature and current clinical understanding of fibrosing ILD behavior in SSc. Therefore, the present findings should not be interpreted as establishing new prognostic associations, but rather as real-world observations consistent with previously reported data.

### 4.3. Radiologic–Functional Dissociation and Persistent Fibrosis

An important observation emerging from this study is the partial dissociation between functional and radiologic outcomes. While pulmonary function parameters remained stable or improved, radiologic progression, as reflected by increasing Warrick scores, was still observed in both NSIP and UIP subgroups. This apparent discrepancy may reflect the complex relationship between functional and structural pulmonary evolution in SSc-ILD. It also raises the possibility that functional stabilization and radiologic progression may reflect different underlying components of SSc-ILD evolution during follow-up.

These observations illustrate the complementary use of functional and radiologic assessment during follow-up.

However, because HRCT evaluations were based on routine clinical reporting and semi-quantitative Warrick scoring without centralized blinded review or formal inter-observer reproducibility assessment, the observed radiologic-functional dissociation should be interpreted cautiously and considered exploratory rather than definitive.

### 4.4. Subsequent Antifibrotic Use Observed During Routine Clinical Care

In our cohort, nintedanib was subsequently initiated in 55% of patients. Patients who subsequently received nintedanib generally exhibited more advanced pulmonary involvement at baseline, including UIP pattern, lower baseline DLCO values, and severe ILD. Because of the strong interrelationship among disease severity variables and the limited cohort size, the present study cannot determine whether these characteristics were independently associated with progression.

The SENSCIS trial and its long-term extension studies demonstrated that nintedanib reduces the rate of FVC decline in patients with SSc-ILD, including those receiving background MMF therapy [8]. Real-world studies have further confirmed the effectiveness and acceptable safety profile of nintedanib, both as monotherapy and in combination with immunosuppressive agents [17,18].

Our findings are consistent with previously reported observations from clinical trials and real-world studies describing treatment pathways observed in routine clinical practice in SSc-ILD, in which MMF is commonly used as first-line therapy, followed by subsequent antifibrotic use in patients with persistent disease progression. However, the present study was not designed to establish causal relationships or to define standardized management approaches according to HRCT pattern.

In particular, although subsequent nintedanib use was more frequently observed among patients with UIP pattern, the small subgroup size and retrospective design substantially limit interpretation of these descriptive observations in routine clinical practice. Larger prospective studies with standardized radiologic and functional assessment are needed to validate these exploratory observations in SSc-ILD.

These observations further illustrate the heterogeneity of pulmonary evolution and therapeutic pathways encountered in routine clinical practice in SSc-ILD.

### 4.5. Comparison with Previous Real-World and Clinical Trial Observations

The study by Fischer et al. (2013) demonstrated that MMF therapy is associated with stabilization or even improvement of pulmonary function, reflected by significant increases in FVC and DLCO, especially in patients without a UIP pattern [19]. In contrast, in the UIP subgroup, the authors primarily reported stabilization of functional parameters without significant improvement. These findings are fully concordant with our observations, in which patients with UIP showed a more modest functional response and a more frequent progression during follow-up compared with those with NSIP.

More recent data reported by Ohta et al. (2025) are also consistent with stabilization or improvement of pulmonary and cutaneous manifestations observed during MMF therapy in patients with systemic sclerosis [20].

An aspect explored in the present cohort was the real-world use of combined immunosuppressive and antifibrotic approaches during follow-up, particularly in patients with progressive fibrosing features. Moreover, our observation was that patients who subsequently received nintedanib generally exhibited UIP-pattern disease and more advanced pulmonary involvement [9,17].

In this context, nintedanib has emerged as a key therapeutic option, supported by randomized clinical trial data demonstrating a significant reduction in the annual rate of FVC decline in patients with SSc-ILD, with sustained efficacy over long-term follow-up [10,21]. Furthermore, real-world studies have confirmed that nintedanib, administered alone or in combination with immunosuppressive agents such as MMF, can contribute to stabilization of lung function, although it does not completely halt disease progression [19,20].

Taken together, these data are consistent with the observations of the present cohort and further illustrate the real-world use of combined immunosuppressive and antifibrotic strategies in patients with progressive SSc-ILD. This observation may be particularly relevant in patients with UIP-pattern disease or evidence of ongoing fibrotic progression, although larger prospective studies are required before clinical observations can be more clearly defined.

Beyond confirming findings previously reported in the literature, the present study provides exploratory real-world observations regarding pulmonary functional and radiologic evolution under MMF therapy in patients with SSc-ILD treated in routine clinical practice.

### 4.6. Clinical Implications

The findings of this study provide exploratory observations regarding routine clinical care of patients with SSc-ILD. However, these observations should be interpreted within the context of the exploratory design, retrospective methodology, and limited sample size of the present study.

First, MMF was associated with stabilization of pulmonary function, with preservation of FVC and improvement in DLCO observed during follow-up. Although these observations are consistent with the established role of MMF in SSc-ILD, the present data are insufficient to establish definitive comparative efficacy across radiologic subgroups.

Second, HRCT patterns may represent potentially relevant imaging features observed during routine clinical assessment and longitudinal monitoring of patients with SSc-ILD. These findings illustrate how functional and radiologic follow-up parameters were integrated into routine clinical care within this cohort, according to baseline HRCT pattern and pulmonary disease severity.

Third, combined functional and radiologic follow-up was routinely performed during routine clinical practice in this cohort. In some patients, radiologic progression was observed despite apparent functional stabilization during follow-up under MMF therapy. However, because radiologic progression in the present study was assessed using routine HRCT reports and semi-quantitative scoring without centralized review, these observations require cautious interpretation.

Finally, the combined use of immunosuppressive and antifibrotic therapies observed in this cohort reflects real-world combined immunosuppressive and antifibrotic approaches used in patients with progressive fibrosing disease. However, because of the exploratory nature of the study and the limited sample size, no definitive conclusions can be drawn regarding patterns of therapeutic use according to HRCT pattern. Larger prospective studies with standardized radiologic assessment are required to further clarify these exploratory observations.

### 4.7. Exploratory Nature of the Findings

Because of the small cohort size, limited subgroup numbers, and retrospective single-center design, all subgroup comparisons and adjusted analyses should be interpreted as exploratory and descriptive rather than confirmatory. The present study was not designed or statistically powered to establish independent associations with progression, treatment response, or management approaches in SSc-ILD.

Accordingly, the findings are intended primarily to describe real-world clinical observations regarding pulmonary functional and radiologic evolution under MMF therapy, while generating hypotheses for future prospective studies with larger and more standardized cohorts.

### 4.8. Study Limitations

Several limitations should be acknowledged. Firstly, the small sample size and limited subgroup numbers substantially reduce the statistical power and generalizability of the findings. Because of the low number of events, the adjusted regression model was intentionally restricted to a limited number of clinically relevant variables in order to reduce the risk of overfitting and preserve model stability. Consequently, the present analyses cannot establish independent associations with progression or subsequent antifibrotic use.

Secondly, the retrospective design may introduce selection and information bias, and the absence of a control group precludes definitive conclusions regarding causality. Therefore, causal relationships between MMF treatment and pulmonary outcomes cannot be definitively established, and the observed functional stabilization should be interpreted as an association observed under MMF therapy rather than as direct proof of treatment efficacy.

An additional important limitation relates to treatment heterogeneity before MMF initiation. A substantial proportion of patients had prior exposure to multiple immunosuppressive agents, including cyclophosphamide and azathioprine, with variable treatment durations and non-standardized transitions between therapies. Consequently, cumulative therapeutic exposure and potential residual effects of previous treatments may have influenced pulmonary function trajectories, radiologic evolution, and subsequent initiation of nintedanib during follow-up. This introduces potential confounding by indication and limits the ability to attribute observed outcomes specifically to MMF therapy alone.

Additionally, subsequent initiation of nintedanib was based on physician decision-making in routine clinical practice rather than a standardized protocol. Therefore, subsequent antifibrotic use may have been influenced not only by disease severity and radiologic progression, but also by physician preference, local prescribing practices, reimbursement policies, and drug availability. Because subsequent initiation of nintedanib was not protocolized, the study is also subject to potential confounding by indication.

Patients who subsequently received nintedanib generally exhibited more advanced pulmonary involvement at baseline, which may have influenced subsequent outcomes independently of treatment exposure.

Another limitation relates to the imbalance in baseline pulmonary severity between HRCT subgroups. Patients with UIP pattern exhibited more advanced radiologic and functional impairment at study entry, including higher Warrick scores and lower DLCO values. Consequently, the study cannot determine whether the greater progression observed in UIP-pattern disease reflects the independent prognostic role of HRCT pattern or simply more severe baseline disease.

An important limitation of the present study relates to radiologic assessment methodology. HRCT evaluation was based on routine clinical reports and semi-quantitative Warrick scoring performed in real-world clinical practice, without centralized or blinded radiologic review. Because of the retrospective real-world design, imaging evaluations were performed by different radiologists involved in routine patient care rather than by a single dedicated reader. As a result, some degree of inter-observer variability and reduced reproducibility cannot be excluded. Moreover, HRCT examinations were not centrally re-evaluated in a blinded manner for research purposes, and formal inter-observer agreement analyses were not available. Consequently, variability in radiologic interpretation and longitudinal Warrick score assessment cannot be fully excluded. This limitation is particularly relevant because radiologic progression represented an exploratory imaging outcome in the present study and was integrated into the assessment of disease evolution and subsequent antifibrotic use.

Furthermore, the use of semi-quantitative scoring may not fully capture subtle longitudinal fibrotic changes compared with standardized centralized imaging analysis or quantitative imaging approaches.

Despite these limitations, the study provides valuable real-world descriptions regarding pulmonary functional and radiologic evolution and therapeutic pathways observed during routine clinical care in SSc-ILD.

### 4.9. What This Study Adds

○ Provides exploratory real-world descriptions of pulmonary functional and radiologic evolution under MMF therapy in patients with SSc-ILD.
○ Describes concomitant functional stabilization and radiologic progression observed during follow-up.
○ Describes differences in radiologic and functional evolution across NSIP and UIP HRCT patterns within a small retrospective cohort.
○ Describes heterogeneous therapeutic pathways observed in routine clinical care, including delayed MMF initiation, prior immunosuppressive exposure, and subsequent antifibrotic use.
○ Describes subsequent antifibrotic use observed during routine clinical care in patients with more advanced pulmonary involvement.

Highlights the need for larger prospective studies with standardized imaging assessment to further evaluate pulmonary evolution in SSc-ILD.

## 5. Conclusions

This small retrospective real-world case series describes pulmonary functional and radiologic evolution in patients with SSc-ILD treated with MMF in routine clinical practice.

Overall functional stabilization was observed during follow-up, while radiologic progression appeared more frequent in patients with UIP-pattern disease and more advanced baseline pulmonary involvement.

Because of the small sample size and retrospective descriptive design, these observations should be considered exploratory and hypothesis-generating only and should not be interpreted as evidence of causal associations, independent predictors, or therapeutic recommendations.

## Figures and Tables

**Table 1:** Baseline characteristics of patients with SSc-ILD.

Characteristic	Value (N = 20)
Demographic characteristics	
Age, years (mean ± SD)	56.4 ± 11.1
Female *n* (%)	15 (75)
Age at SSc onset, years (mean ± SD)	46.3 ± 13.2
Age at SSc-ILD diagnosis, years (mean ± SD)	47.6 ± 9.5
Disease characteristics	
SSc subtype, *n* (%)	
Limited cutaneous SSc, *n* (%)	4 (20)
Diffuse cutaneous SSc, *n* (%)	13(65)
SSc sine scleroderma	3 (15)
Nailfold capillaroscopy pattern	
Early pattern, *n* (%)	2 (10)
Active pattern, *n* (%)	13 (65)
Late pattern, *n* (%)	3 (15)
Not available/non-specific	2 (10)
Serological profile	
Anti-Scl-70 positive, *n* (%)	17 (85)
Anticentromere positive, n (%)	3 (15)
Pulmonary characteristics	
NSIP pattern, *n* (%)	13 (65)
UIP pattern, *n* (%)	7 (35)
FVC (% predicted, mean ± SD)	91.2 ± 11.3
DLCO (% predicted, mean ± SD)	31.4 ± 20.4
Dyspnea, *n* (%)	17 (85)
Medsger pulmonary score	
Mild (0–1) *n* (%)	3 (15)
Moderate (2) *n* (%)	6 (30)
Severe (3–4) *n* (%)	11 (55)
Organ involvement	
Skin involvement, *n* (%)	15 (75)

**Table 2:** Evolution of pulmonary function parameters under MMF treatment.

Parameter	Comparison	Mean Difference	95% CI	*p*-Value
FVC (%)	Baseline vs. 6 months	+0.8	−4.34 to 5.94	0.748
	6 vs. 12 months	+0.7	−6.34 to 7.73	0.834
	Baseline vs. 12 months	+6.3	−0.06 to 12.68	0.052
DLCO (%)	Baseline vs. 6 months	+5.8	0.07 to 11.58	0.048
	6 vs. 12 months	+3.1	−2.72 to 8.86	0.272
	Baseline vs. 12 months	+9.0	1.61 to 16.39	0.021

Mean differences represent paired within-group changes between time points. CI, confidence interval.

**Table 3:** (**a**). Descriptive clinical characteristics of patients according to subsequent nintedanib use. (**b**). Exploratory adjusted logistic regression analysis for subsequent initiation of nintedanib.

(**a**)			
**Variable**	**Nintedanib (*n* = 11)**	**No Nintedanib (*n* = 9)**	***p*** **-Value**
Age, years (mean ± SD)	58 ± 10	53 ± 11	0.28
Female, *n* (%)	8 (72.7)	7 (77.8)	1.00
Disease duration, years (median)	10	3	0.06
FVC (% predicted, median [IQR])	85 (84–95)	95 (88–100)	0.20
DLCO (% predicted, median [IQR])	40 (35–40)	60 (55–60)	0.04
Dyspnea, n (%)	10 (90.9)	7 (77.8)	0.58
UIP pattern, n (%)	7 (63.6)	0 (0)	0.01
NSIP pattern, n (%)	4 (36.4)	9 (100)	0.01
Diffuse cutaneous SSc, *n* (%)	9 (81.8)	4 (44.4)	0.09
Severe ILD (DLCO < 40%), *n* (%)	6 (54.5)	1 (11.1)	0.03
Prior immunosuppressive exposure before MMF, *n* (%)	9 (81.8)	4 (44.4)	0.09
(**b**)			
**Variable**	**Adjusted OR**	**95% CI**	***p*** **-Value**
UIP pattern	3.23	0.41–25.23	0.263
Baseline DLCO	0.97	0.91–1.03	0.297

OR, odds ratio; CI, confidence interval. Estimates should be interpreted cautiously given the limited sample size and low number of events.

**Table 4:** Treatment initiation characteristics and prior therapeutic exposure before MMF in SSc-ILD.

Variable	Value
MMF as first-line therapy	7 (35%)
MMF as second-line or subsequent therapy	13 (65%)
Time from diagnosis to MMF initiation	
– Mean	4.9 years
– Median (IQR)	3 (0.85–6.5) years
– Range	0.5–18 years
Time categories	
<1 year	5 (25%)
1–5 years	8 (40%)
>5 years	7 (35%)
Prior therapies before MMF	
Cyclophosphamide	3 (15%)
Azathioprine	4 (20%)
Methotrexate	5 (25%)
Hydroxychloroquine	5 (25%)
Vasodilator therapy	12 (60%)

**Table 5:** Descriptive Characteristics According to MMF Treatment Timing in patients with SSc-ILD.

Variable	First-Line MMF (*n* = 7)	Subsequent MMF (*n* = 13)	*p*-Value
Age, years (mean ± SD)	55.0 ± 8.8	57.4 ± 11.6	0.63
Female sex, *n* (%)	4 (57.1)	11 (84.6)	0.38
Disease duration at MMF initiation, years (mean ± SD)	1.8 ± 2.3	10.7 ± 15.8	0.09
FVC (% predicted, mean ± SD)	84.9 ± 25.7	88.7 ± 19.7	0.68
DLCO (% predicted, mean ± SD)	44.1 ± 20.7	46.6 ± 19.5	0.74
Dyspnea, *n* (%)	6 (85.7)	11 (84.6)	1.00
UIP pattern, *n* (%)	2 (28.6)	5 (38.5)	0.63
NSIP pattern, *n* (%)	5 (71.4)	8(61.5)	0.63
Diffuse cutaneous SSc, *n* (%)	2 (28.6)	11 (84.6)	0.03
Severe ILD (DLCO < 40%), *n* (%)	2 (28.6)	5 (38.5)	0.64

## Data Availability

The data presented in this study are available on request from the corresponding author due to privacy and ethical restrictions related to anonymized patient clinical and imaging data.

## Abbreviations

ACR/EULAR	American College of Rheumatology/European Alliance of Associations for Rheumatology (classification criteria)
ATS/ERS/JRS/ALAT	American Thoracic Society/European Respiratory Society/Japanese Respiratory Society/Asociación Latinoamericana de Tórax
DLCO	Diffusing Capacity for Carbon Monoxide
eGFR	Estimated Glomerular Filtration Rate
EULAR	European Alliance of Associations for Rheumatology
FVC	Forced Vital Capacity
GERD	Gastroesophageal Reflux Disease
GI	Gastrointestinal
GGO	Ground-Glass Opacities
HRCT	High-Resolution Computed Tomography
ILD	Interstitial Lung Disease
IPF	Idiopathic Pulmonary Fibrosis
MD	Mean Difference
MMF	Mycophenolate Mofetil
MPA	Mycophenolic Acid
NSIP	Nonspecific Interstitial Pneumonia
PFT/PFTs	Pulmonary Function Test(s)
RR	Risk Ratio
SD	Standard Deviation
SSc	Systemic Sclerosis
SSc-ILD	Systemic Sclerosis-Associated Interstitial Lung Disease
TDM	Therapeutic Drug Monitoring
UIP	Usual Interstitial Pneumonia
